# Akt and mitogen‐activated protein kinase enhance C‐type lectin‐like receptor 2‐mediated platelet activation by inhibition of glycogen synthase kinase 3α/β

**DOI:** 10.1111/jth.12954

**Published:** 2015-05-09

**Authors:** A. J. Moroi, S. P. Watson

**Affiliations:** ^1^ Centre for Cardiovascular ScienceCollege of Medical and Dental SciencesUniversity of BirminghamBirminghamUK

**Keywords:** C‐type lectin, glycogen synthase kinase 3, mitogen‐activated protein kinases, phosphatidylinositol 3‐kinases, protein kinase B

## Abstract

**Background:**

The C‐type lectin‐like receptor 2 (CLEC‐2) and the collagen receptor glycoprotein (GP)VI activate platelets through Src and Syk tyrosine kinases, and phospholipase Cγ2. The initial events in the two signaling cascades, however, are distinct, and there are quantitative differences in the roles of proteins downstream of Syk activation. The activation of Akt and mitogen‐activated protein kinases (MAPKs) has been shown to enhance platelet activation by GPVI, but their role in CLEC‐2 signaling is not known.

**Objectives:**

We sought to investigate the role of the Akt and MAPK pathways in platelet activation by CLEC‐2.

**Results:**

The CLEC‐2 agonist rhodocytin stimulated phosphorylation of Akt and p38 and extracellular signal‐related kinase (ERK) MAPKs, but with a delay relative to Syk. Phosphorylation of these proteins was markedly inhibited in the combined presence of apyrase and indomethacin, consistent with the reported feedback action of ADP and thromboxane A_2_ in CLEC‐2 signaling. Phosphorylation of Akt and phosphorylation of ERK were blocked by the phosphoinositide 3‐kinase (PI3K) inhibitor wortmannin and the protein kinase C (PKC) inhibitor Ro31‐8220, respectively, whereas Syk phosphorylation was not altered. On the other hand, both inhibitors reduced phosphorylation of the Akt substrate glycogen synthase kinase 3α/β (GSK3α/β). Phosphorylation of GSK3α/β was also blocked by the Akt inhibitor MK2206, and reduced at late, but not early, times by the MEK inhibitor PD0325901. MK2206 and PD0325901 inhibited aggregation and secretion in response to a low concentration of rhodocytin, which was restored by GSK3α/β inhibitors.

**Conclusions:**

These results demonstrate that CLEC‐2 regulates Akt and MAPK downstream of PI3K and PKC, leading to phosphorylation and inhibition of GSK3α/β, and enhanced platelet aggregation and secretion.

## Introduction

C‐type lectin‐like receptor 2 (CLEC‐2) is a C‐type lectin‐like receptor that is highly expressed in platelets and expressed at a low level in other hematopoietic cells 1, 2, 3. The only known endogenous ligand for CLEC‐2 is podoplanin, a sialomucin‐like glycoprotein that is expressed in a wide variety of cells outside of the vasculature 4. CLEC‐2 is also activated by the snake venom toxin rhodocytin.

CLEC‐2 signals through a similar pathway to the collagen receptor gylcoprotein (GP)VI/FcRγ, which utilizes an immunoreceptor tyrosine‐based activation motif (ITAM) in the cytosolic tail of FcRγ 5. An ITAM has two conserved YxxL sequences separated by 6–12 amino acids that, when phosphorylated, bind to the tandem SH2 domains in Syk, inducing kinase activation. CLEC‐2 has a single YxxL motif, known as a hemITAM, which activates Syk through the bridging of two phosphorylated receptors via its tandem SH2 domains. In contrast to GPVI, the activation of CLEC‐2 in human platelets is critically dependent on the feedback actions of ADP and thromboxane A_2_ (TxA_2_), and actin polymerization 6.

The activation of Syk by CLEC‐2 or GPVI initiates a signaling cascade of adapter and effector proteins that culminates in the activation of phospholipase Cγ2 (PLCγ2) 7, 8, 9. This signaling cascade is conserved between the two receptors, although a number of quantitative and qualitative differences have been reported, including the ability of high concentrations of CLEC‐2 but not of GPVI agonists to activate platelets deficient in the adapter protein SLP‐76 10.

The phosphoinositide 3‐kinase (PI3K)–Akt pathway has been shown to support platelet activation by GPVI through the regulation of the serine/threonine kinase Akt (also known as protein kinase B) 11. Mouse and human platelets express all three known isoforms of Akt, i.e. Akt1, Akt2, and Akt3, and all have been shown to contribute to platelet activation 12, 13, 14, 15, 16. Akt family proteins are regulated through phosphorylation of Thr308 and Ser473 by phosphoinositide‐dependent kinase 1 (PDK1) and mammalian target of rapamycin complex 2, respectively 12, 17, 18, 19, 20. The generation of phosphatidylinositol 3,4,5‐trisphosphate by PI3K recruits PDK1 and Akt to the plasma membrane via their pleckstrin homology (PH) domains, leading to PDK1‐dependent phosphorylation of Akt. In addition, Akt can be phosphorylated by protein kinase C (PKC) and by Ca^2+^/calmodulin‐dependent protein kinase kinase (CaMKK) independently of PI3K 21, 22, 23. Activation by collagen is impaired in mouse platelets deficient in Akt1 16, possibly as a consequence of loss of phosphorylation of the inhibitory protein glycogen synthase kinase 3α/β (GSK3α/β) 24. However, inhibition of GSK3α/β in human platelets has no effect on activation by the synthetic GPVI agonist collagen‐related peptide (CRP), challenging this mechanism 13.

Mitogen‐activated protein kinases (MAPKs) are divided into four subgroups: extracellular signal‐related kinases (ERK), c‐Jun N‐terminal kinases (JNK), big mitogen‐activated protein kinase 1 (BMK1; ERK5), and p38. Among these, ERK, JNK and p38 are expressed in platelets, and are regulated by a wide range of receptors. ERK is activated in response to collagen, and the MEK inhibitor U0126 blocks activation by low concentrations of collagen 25, 26. On the other hand, Börsch‐Haubold *et al*. 27 demonstrated that the MEK inhibitor PD98059 had no effect on platelet aggregation induced by collagen, although this study was performed in the presence of a cyclooxygenase inhibitor, as PD98059 also inhibits this class of enzyme 28. These results therefore suggest that ERK may contribute to platelet activation by GPVI through the regulation of thromboxane formation.

In this study, we investigated the role of the PI3K–Akt and MAPK pathways in platelet activation by CLEC‐2 by using the pharmacologic inhibitors MK2206 and PD0325901, which block Akt and the MAPK‐activating protein MEK, respectively. We show that Akt and ERK support platelet aggregation in response to low concentrations of the CLEC‐2‐activating snake toxin rhodocytin through phosphorylation of GSK3α/β, thus preventing its inhibitory action. Moreover, the PKC inhibitor Ro31‐8220 also reduces GSK3α/β phosphorylation. These results demonstrate that CLEC‐2 regulates GSK3α/β phosphorylation downstream of Akt, ERK, and PKC, thus enhancing platelet activation.

## Materials and methods

### Reagents and antibodies

Rhodocytin was purified from *Calloselasma rhodostoma* venom as previously described 29. Horm collagen was from Takeda (Munich, Germany). Crosslinked CRP was from R. Farndale (Cambridge University, UK). The anti‐phosphotyrosine mAb 4G10 was from Upstate Biotechnology (TCS Biologicals, Buckingham, UK). Anti‐phospho‐Akt (Thr308), anti‐phospho‐p38 (Thr180/182), anti‐phospho‐Syk (Tyr352), anti‐phospho‐PLCγ2 (Tyr1217) and anti‐phospho‐GSK3α/β (Ser21/9) were from Cell Signaling Technology (New England Biolabs, Hitchin, UK). Anti‐Syk, anti‐phospho‐ERK1/2 (Thr202/Tyr204) and anti‐ERK2 were from Santa Cruz Biotechnology (Heidelberg, Germany). MK2206, CHIR‐99021 and PD0325901 were from Selleck Chemicals (Stratech, Newmarket, UK). PRT‐318 was provided by Portola Pharmaceuticals (San Francisco, CA, USA). All other reagents were from Sigma‐Aldrich (Poole, UK) or from previously named sources 30.

### Platelet preparation

All donors gave informed consent, and the study was approved by the University of Birmingham ethical review committee. Platelet preparation was performed as previously described 31. Venous blood from healthy drug‐free volunteers was taken into 10% sodium citrate, and mixed with 1 : 9 (v/v) acid citrate dextrose (120 mm sodium citrate, 110 mm glucose, and 80 mm citric acid), and centrifuged at 200 × *g* to obtain platelet‐rich plasma (PRP). Prostacyclin (0.5 μg mL^−1^) was added, and PRP was centrifuged at 1000 × *g* for 10 min to obtain a platelet pellet. The platelets were washed once by resuspension in HEPES–Tyrode's buffer (134 mm NaCl, 2.9 mm KCl, 0.34 mm Na_2_HPO_4_.12H_2_O, 12 mm NaHCO_3_, 20 mm HEPES, 1 mm MgCl_2_, and 5.0 mm glucose [pH 7.3]) and further centrifugation at 1000 × *g* for 10 min in the presence of prostacyclin (0.5 μg mL^−1^) and 1 : 9 (v/v) acid citrate dextrose. The pellet of washed platelets was resuspended in a small volume of the HEPES–Tyrode's buffer, and then diluted to an appropriate concentration for experimentation: a cell density of 2 × 10^8^ mL^−1^ was used for aggregation, and a cell density of 5 × 10^8^ mL^−1^ was used for western blotting.

### Western blotting

To inhibit aggregation, washed platelets were pretreated with 9 μm integrilin (eptifibatide), unless otherwise mentioned. Samples of washed platelets (300 μL) were stimulated with rhodocytin in an aggregometer at 1200 r.p.m. and 37 °C. Platelets were pretreated for 15 min with the following inhibitors (final concentrations indicated in parentheses): apyrase (2 U mL^−1^), indomethacin (10 μm), PRT‐318 (5 μm), PP2 (10 μm), BAPTA‐AM (10 μm), wortmannin (100 nm), Ro31‐8220 (5 μm), LY294002 (5 μm), MK2206 (1 μm), and PD0325901 (5 μm). An equal concentration of dimethylsulfoxide (0.2%) was added to the controls. Reactions were terminated by addition of an equal volume of ice‐cold 2 × lysis buffer (300 mm NaCl, 20 mm Tris, 2 mm EGTA, 2 mm EDTA, and 2% NP40 [pH 7.5]). The samples were diluted with an equal volume of 2 × sample buffer (4% SDS, 10% 2‐mercaptoethanol, 20% glycerol, and 50 mm Tris [pH 6.8]), separated by SDS‐PAGE (10%), and transferred to a poly(vinylidene difluoride) membrane. Western blotting was performed with the indicated antibodies. Densitometry of the bands was performed with image j software (NIH, Bethesda, MD, USA).

### Platelet aggregation and ATP secretion

Aggregation was monitored by light transmission with a Born lumi‐aggregometer (Chronolog, Harvertown, PA, USA). ATP secretion was measured with a luciferin/luciferase substrate/enzyme mix (Chronolume).

### Statistics

All experiments were performed three to five times, and data are shown as means ± standard errors of the mean. Statistical analysis was performed with one‐way anova followed by the Newman–Keuls test. A *P*‐value of < 0.05 defined significant differences between test groups.

## Results

### The PI3K–Akt and MAPK pathways are activated by CLEC‐2

**Figure 1:**
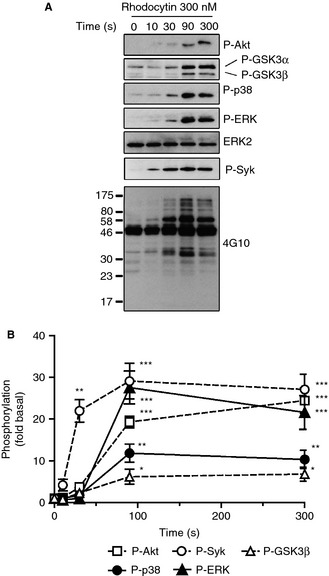
The phosphoinositide 3‐kinase–Akt and mitogen‐activated protein kinase pathways are activated after C‐type lectin‐like receptor 2 stimulation. (A) Washed human platelets (5 × 10^8^ mL^−1^) were pretreated with integrilin (9 μm). Platelets were then preincubated with the vehicle (dimethylsulfoxide [DMSO]), and stimulated with 300 nm rhodocytin for the indicated times before extraction. Whole cell lysates were analyzed for phosphorylation of Akt (Thr308), p38 (Thr180/182), extracellular signal‐related kinase (ERK) (Thr202/Tyr204), glycogen synthase kinase 3α/β (GSK3α/β) (Ser21/9) and Syk (Tyr352) by western blotting. Total tyrosine phosphorylation was detected with 4G10 mAb, and ERK2 was used as a loading control. (B) Densitometric measurements of phospho‐Akt, phospho‐p38, phospho‐ERK, phospho‐Syk and phospho‐GSK3β are expressed as fold change over the basal control (DMSO only, no activation). The figure is representative of three independent experiments. Data are reported as the mean ± standard error of the mean (*n* = 3). *P <* *0.05, **P <* *0.01 and ***P <* *0.001 as compared with the basal phosphorylation level of each molecule.

In the present study, we investigated the role of the PI3K–Akt and MAPK pathways in platelet activation by CLEC‐2. As shown in Fig. 1, rhodocytin stimulated phosphorylation of Akt, and p38 and ERK MAPKs, with a delay of 30–90 s (Fig. 1A,B). Phosphorylation of the Akt substrate, GSK3α/β, at its negative regulatory site occurred in parallel with phosphorylation of Akt (Fig. 1A,B). In contrast, robust phosphorylation of Syk at the activation tyrosine, Tyr352 32, occurred within 10–30 s (Fig. 1A,B), suggesting that this lies upstream of phosphorylation of the Akt and MAPK pathways. Consistent with this, phosphorylation of Akt and MAPK, as well as of PLCγ2, which has been previously shown to be regulated downstream of Syk, by rhodocytin was blocked by the Syk inhibitor PRT‐318 and the Src inhibitor PP2 (Fig. S1A). Activation of the collagen receptor GPVI by CRP was also associated with a delay in phosphorylation of Akt, p38 and ERK relative to Syk (Fig. S2), as previously reported 33, 34. Phosphorylation of Akt and MAPK by rhodocytin was maintained in the presence of the α_IIb_β_3_ blocker integrilin, demonstrating that phosphorylation is independent of outside‐in signaling by the integrin (Fig. S1A).

**Figure 2:**
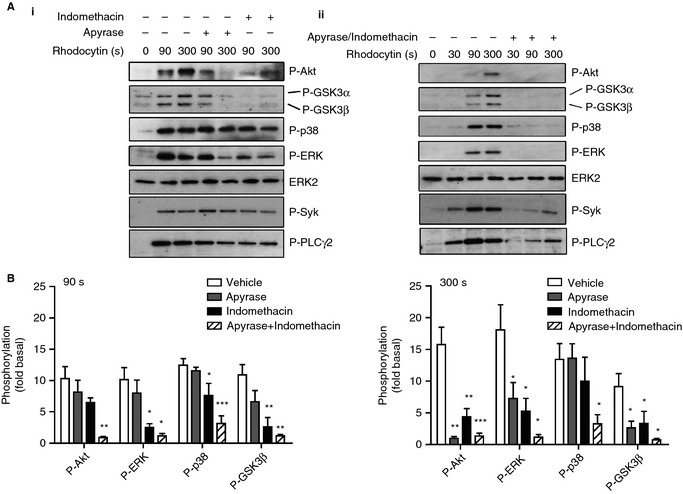
Role of secondary mediators in C‐type lectin‐like receptor 2‐mediated Akt and mitogen‐activated protein kinase activation. Washed human platelets (5 × 10^8^ mL^−1^) were pretreated with integrilin (9 μm). Platelets were then preincubated with 2 U mL^−1^ apyrase or 10 μm indomethacin (AI), apyrase + indomethacin (AII), or dimethylsulfoxide (DMSO). Platelets were stimulated with 300 nm rhodocytin for the indicated times. Whole cell lysates were analyzed for phosphorylation of Akt (Thr308), p38 (Thr180/182), extracellular signal‐related kinase (ERK) (Thr202/Tyr204), glycogen synthase kinase 3α/β (GSK3α/β) (Ser21/9), Syk (Tyr352), and phospholipase Cγ2 (PLCγ2) (Tyr1217). ERK2 was used as a loading control. (B) Densitometric measurements of phospho‐Akt, phospho‐p38, phospho‐ERK and phospho‐GSK3β at 90 s (left) and 300 s (right) after stimulation are expressed as fold increase over the basal level (DMSO, no rhodocytin). The blot shown is representative of three independent experiments. Data are reported as the mean ± standard error of the mean (*n* = 3). *P <* *0.05, **P <* *0.01 and ***P <* *0.001 as compared with the vehicle control.

We have previously reported that the secondary agonists ADP and TxA_2_ play a critical feedback role in the regulation of CLEC‐2 signaling in human platelets 6. In line with this, the ATP scavenger apyrase and the cyclooxygenase inhibitor indomethacin reduced phosphorylation of Akt and ERK by rhodocytin (300 nm), whereas phosphorylation of p38 was only slightly inhibited (Fig. 2AI,B). In contrast, phosphorylation of Akt and ERK, as well as of p38, was abolished in the presence of the combination of indomethacin and apyrase, and phosphorylation of Syk and PLCγ2 was markedly reduced (Fig. 2B). These results are consistent with a pathway in which activation of CLEC‐2 and Syk by rhodocytin is reinforced by ADP and TxA_2_, as previously reported 6, leading to robust phosphorylation of Akt, ERK, and p38 (Fig. 2AII,B). The presence of a residual level of phosphorylation of Syk and PLCγ2, in contrast to the complete blockade of Akt, ERK and p38 phosphorylation, suggests that phosphorylation of the latter three proteins is mediated predominantly by the combination of ADP and TxA_2_.

### Akt and ERK are phosphorylated downstream of PI3K and PKC, respectively

**Figure 3:**
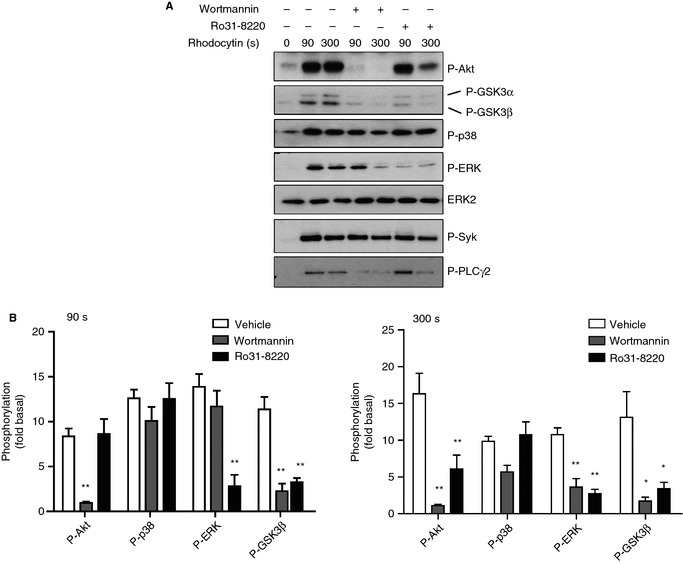
The effects of phosphoinositide 3‐kinase and protein kinase C inhibition on C‐type lectin‐like receptor 2 (CLEC‐2)‐mediated Akt and mitogen‐activated protein kinase phosphorylation. (A) Washed human platelets (5 × 10^8^ mL^−1^) were pretreated with integrilin (9 μm). Platelets were then preincubated with 100 nm wortmannin, 5 μm Ro31‐8220, or dimethylsulfoxide (DMSO). Platelets were stimulated with 300 nm rhodocytin for the indicated times. Whole cell lysates were analyzed for phosphorylation of Akt (Thr308), p38 (Thr180/182), extracellular signal‐related kinase (ERK) (Thr202/Tyr204), glycogen synthase kinase 3α/β (GSK3α/β) (Ser21/9), Syk (Tyr352), and phospholipase Cγ2 (PLCγ2) (Tyr1217). ERK2 was used as a loading control. (B) Densitometric measurements of phospho‐Akt, phospho‐p38, phospho‐ERK and phospho‐GSK3β at 90 s (left) and 300 s (right) after stimulation are expressed as fold increase over the basal control (DMSO, or rhodocytin). The blot shown is a representative of three independent experiments. Data are reported as the mean ± standard error of the mean (*n* = 3). *P <* *0.05 and **P <* *0.01 as compared with the vehicle control.

We used pharmacologic inhibitors to further investigate the regulation of Akt and MAPK by CLEC‐2. The PI3K inhibitor wortmannin abolished Akt phosphorylation by CLEC‐2 and inhibited phosphorylation of ERK at later time points, but had a minimal effect on phosphorylation of Syk and of p38 MAPK (Fig. 3A,B). On the other hand, PLCγ2 activation was dramatically reduced in the presence of wortmannin (Fig. 3A). In addition, wortmannin inhibited phosphorylation of GSK3α/β (Fig. 3A,B). Similar results were obtained with a structurally distinct PI3K inhibitor, LY294002 (data not shown).

In comparison, the PKC inhibitor Ro31‐8220 blocked phosphorylation of ERK at early and later times, but had no effect on phosphorylation of p38 or Syk (Fig. 3A,B). In addition, Ro31‐8220 inhibited phosphorylation of Akt and GSK3α/β at 300 s (Fig. 3A,B). A similar result was obtained with apyrase‐treated platelets (Fig. 2AI,B), suggesting that loss of phosphorylation could be secondary to inhibition of ADP secretion. On the other hand, at 90 s, Ro31‐8220 blocked phosphorylation of GSK3α/β but not of Akt (Fig. 3A,B), thereby dissociating GSK3α/β and Akt phosphorylation, as previously shown in thrombin‐stimulated platelets 13. Phosphorylation of GSK3α/β and Akt was minimally restored at 300 s in the presence of ADP, indicating that it could be partially mediated by loss of ADP secretion, although this did not reach significance (Fig. S1B,C). However, ADP had no effect on GSK3α/β phosphorylation at 90 s. These results therefore suggest that GSK3α/β phosphorylation is mediated both directly and indirectly (through ADP secretion) by PKC, with PKC having the predominant role. BAPTA‐AM had no effect on Akt and MAPK phosphorylation (Fig. S1A) at a concentration that has been shown to block Ca^2+^ elevation at this platelet density 35, 36. This shows that CaMKK is dispensable for phosphorylation.

**Figure 4:**
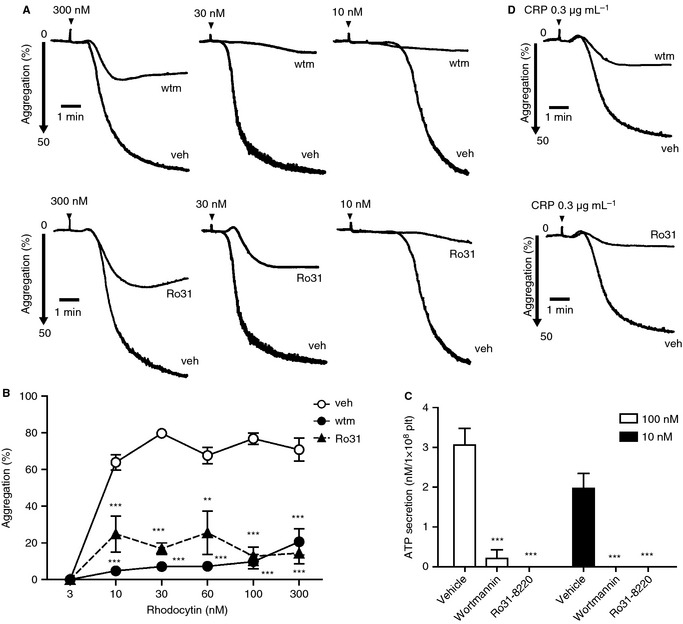
The effects of phosphoinositide 3‐kinase and protein kinase C inhibition on C‐type lectin‐like receptor 2‐mediated platelet secretion and aggregation. (A–D) Washed human platelets (2 × 10^8^ mL^−1^) preincubated with 100 nm wortmannin, 5 μm Ro31‐8220 or dimethylsulfoxide (DMSO) (vehicle control) were stimulated at 37 °C with the indicated dose of rhodocytin (A–C) or collagen‐related peptide (CRP) (D), and platelet aggregation (A, D) and ATP secretion (C) were measured for 5 min. Each aggregation tracing is representative of results obtained from three to five different donors and experiments. (B) Aggregation (mean ± standard error of the mean [SEM], *n* = 3–5) at 5 min in the presence or absence of wortmannin or Ro31‐8220 was plotted as a function of rhodocytin concentration. (C) Quantification of peak platelet ATP release (mean ± SEM, *n* = 3–5) in the presence of the vehicle, wortmannin or Ro31‐8220 in platelets activated with 10 nm or 100 nm rhodocytin. **P <* *0.01 and ***P <* *0.001 as compared with the vehicle control (DMSO). plt, platelets; Ro31, Ro31‐8220; veh, vehicle; wtm, wortmannin.

Platelet aggregation and secretion induced by all concentrations of rhodocytin were markedly inhibited by wortmannin and Ro31‐8220 (Fig. 4A–C). GPVI‐mediated platelet activation was similarly inhibited by wortmannin or Ro31‐8220 (Fig. 4D; Fig. S3A).

These results demonstrate that Akt and ERK are regulated downstream of PI3K and PKC, respectively, by CLEC‐2, and that both pathways are required for efficient GSK3α/β phosphorylation. PI3K and PKC play a critical role in platelet activation by all concentrations of rhodocytin.

### Akt and ERK enhance platelet activation by CLEC‐2

**Figure 5:**
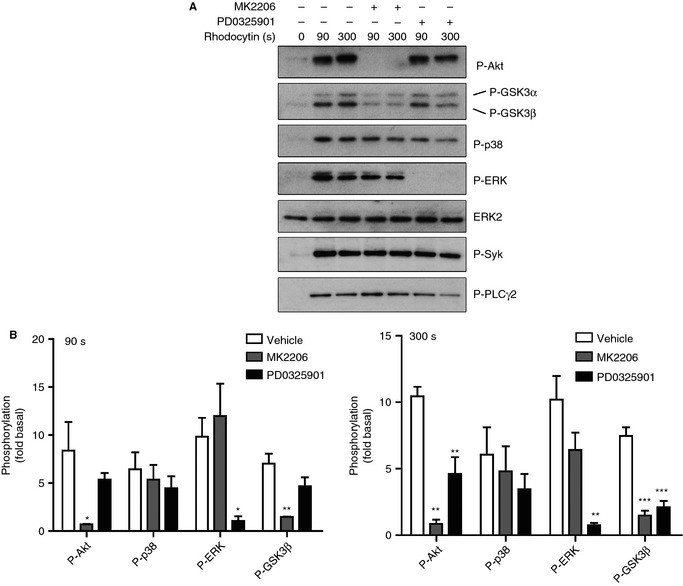
The effects of Akt and extracellular signal‐related kinase (ERK) inhibition on C‐type lectin‐like receptor 2‐mediated glycogen synthase kinase 3α/β (GSK3α/β) phosphorylation. (A) Washed human platelets (5 × 10^8^ mL^−1^) were pretreated with integrilin (9 μm). Platelets were then preincubated with 1 μm MK2206, 5 μm PD0325901 or dimethylsulfoxide (DMSO) (vehicle control). Platelets were stimulated with 300 nm rhodocytin for the indicated times. Whole cell lysate were analyzed for phosphorylation of Akt (Thr308), p38 (Thr180/182), ERK (Thr202/Tyr204), GSK3α/β (Ser21/9), Syk (Tyr352), and phospholipase Cγ2 (PLCγ2) (Tyr1217). ERK2 was used as a loading control. (B) Densitometric measurements of phospho‐Akt, phospho‐p38, phospho‐ERK and phospho‐GSK3β at 90 s (left) and 300 s (right) after stimulation are expressed as fold increase over the basal control (DMSO, no rhodocytin). The blot shown is representative of three independent experiments. **P <* *0.01 and ***P <* *0.001 as compared with the vehicle control.

We used selective inhibitors of the Akt and ERK pathways, MK2206 and PD0325901, respectively, to investigate their role in platelet activation by CLEC‐2. MK2206 binds to the PH domain of Akt, thereby inhibiting its recruitment to the plasma membrane, and preventing phosphorylation by PDK1. In line with this, MK2206 blocked phosphorylation of Akt and reduced phosphorylation of GSK3α/β at 90 s and 300 s (Fig. 5A,B). In contrast, p38 phosphorylation was maintained in the presence of MK2206 (Fig. 5A,B). Inhibition of ERK by PD0325901 inhibited phosphorylation of Akt and GSK3α/β at 300 s (Fig. 5A,B). Thus, ERK mediates delayed phosphorylation of Akt and GSK3α/β. Phosphorylation of Syk and PLCγ2 was maintained in the presence of MK2206 and PD0325901, confirming that activation of Akt and ERK lies downstream of Syk and PLCγ2 (Fig. 5A).

**Figure 6:**
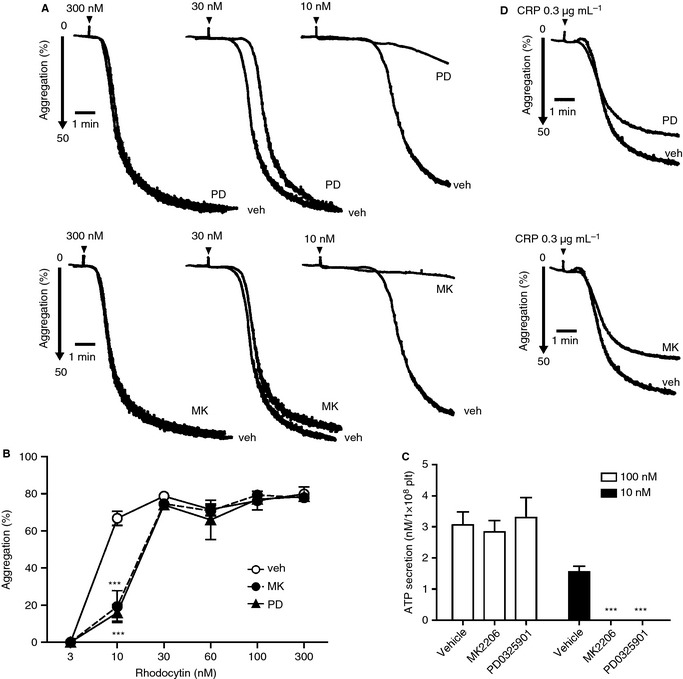
The effect of Akt and mitogen‐activated protein kinase inhibition on C‐type lectin‐like receptor 2‐mediated platelet secretion and aggregation. (A–D) Washed human platelets (2 × 10^8^ mL ^−1^) were preincubated with 1 μm MK2206, 5 μm PD0325901, or dimethylsulfoxide (DMSO) (vehicle control), and then stimulated with the indicated dose of rhodocytin (A–C) and collagen‐related peptide (CRP) (D) at 37 °C; platelet aggregation (A, D) and ATP secretion (C) were then measured for 5 min. Each aggregometer tracing is representative of results obtained from three to five different donors and experiments. (B) Aggregation (mean ± standard error of the mean [SEM], *n* = 3–5) at 5 min in the presence or absence of MK2206 or PD0325901 was plotted as a function of rhodocytin concentration. (C) Quantification of peak platelet ATP release (mean ± SEM, *n* = 3–5) in the presence of the vehicle, MK2206 or PD0325901 in platelets activated with 10 nm or 100 nm rhodocytin. ***P <* *0.001 as compared with the vehicle control (DMSO). MK, MK2206; PD, PD0325901; veh, vehicle.

To investigate the functional significance of the Akt and MAPK pathways, we monitored platelet aggregation and secretion. MK2206 blocked aggregation and ATP secretion induced by a low concentration of rhodocytin (10 nm), and very slightly delayed the onset of aggregation in response to an intermediate concentration (30 nm). There was no inhibition of aggregation or secretion in response to 300 nm rhodocytin (Fig. 6A–C). The MEK inhibitor PD0325901 had a similar effect, with inhibition of aggregation at a low concentration of rhodocytin (10 nm), a slight delay in onset at an intermediate concentration (30 nm), and no effect at a higher concentration (Fig. 6A,B). ATP secretion was also inhibited by PD0325901 in response to a low but not a high concentration of rhodocytin (Fig. 6C). The start of aggregation at a low concentration of rhodocytin (10 nm) was delayed relative to phosphorylation of Akt, ERK, and GSK3α/β, which occurred after 90 s (Fig. 5A), further suggesting that Akt and ERK regulate aggregation. Aggregation induced by a low (0.3 μg mL^−1^) but not a high (10 μg mL^−1^) concentration of CRP was also partially reduced in the presence of MK2206 and PD0325901 (Fig. 6D; Fig. S3B). Together, these results demonstrate that Akt and ERK play a role in platelet activation induced by low concentrations of CLEC‐2 and GPVI receptor agonists.

### GSK3α/β is the downstream effector for the PI3K–Akt and MAPK pathways

**Figure 7:**
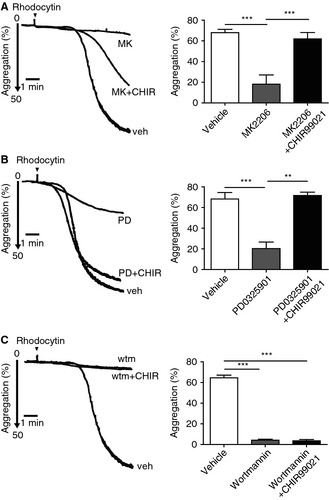
Glycogen synthase kinase 3α/β is the downstream effector for Akt and mitogen‐activated protein kinase activation. Washed human platelets (2 × 10^8^ mL ^−1^) were preincubated with MK2206 (A), 5 μm PD0325901 (B), 100 nm wortmannin (C) or dimethylsulfoxide (vehicle control) in the presence or absence of CHIR‐99021, and stimulated with 10 nm rhodocytin at 37 °C; and platelet aggregation was then measured for 5 min. The left‐side figures show the aggregometer tracings, each being representative of results obtained from three different donors and experiments. The right‐side graphs shows the effect of wortmannin, MK2206 or PD0325901 on aggregation at 5 min in the absence or presence of CHIR‐99021; data bars are each the mean ± standard error of the mean of three experiments. **P <* *0.01 and ***P <* *0.001 as compared with the vehicle control. CHIR, CHIR‐99021; MK, MK2206; PD, PD0325901; veh, vehicle; wtm, wortmannin.

The above results are consistent with a model in which rhodocytin stimulates GSK3α/β phosphorylation at its negative regulatory site downstream of Akt and ERK, thereby inhibiting GSK3α/β activity. To investigate the consequence of loss of phosphorylation of GSK3α/β on platelet activation, we treated platelets with the GSK3α/β inhibitors CHIR‐99021 and SB216763. These are ATP competitive inhibitors that block GSK3 activation but not its phosphorylation. Consistent with the inhibitory action of GSK3α/β, both inhibitors caused a small potentiation of aggregation by rhodocytin (Fig. S4A). Furthermore, inhibition of rhodocytin‐induced platelet aggregation and secretion by MK2206 was reversed in the presence of CHIR‐99021 (Fig. 7A; Fig. S4C) and SB216763 (Fig. S4B). CHIR‐99021 and SB216763 also rescued aggregation and secretion of PD0325901‐treated platelets (Fig. 7B; Fig. S4B,C). In contrast, inhibition of platelet aggregation or secretion after wortmannin treatment was not rescued by CHIR‐99021 (Fig. 7C; Fig. S4C). This indicates that PI3K regulates platelet activation through one or more additional pathways to that of regulation of GSK3α/β. Interestingly, we did not observe potentiation of platelet aggregation by low concentrations of CRP (0.1 μg mL^−1^ and 0.3 μg mL^−1^) in the presence of CHIR‐99021 or SB216763 (Fig. S5A), but observed inhibition of the response to collagen (Fig. S5B), as previously reported 15, 37.

To verify that GSK3α/β is regulated downstream of Akt and ERK in CLEC‐2 signaling independently of α_IIb_β_3_, we treated platelets with integrilin before stimulation and monitored ATP secretion. Rhodocytin‐induced secretion was blocked in the presence of Akt and MEK inhibitors, and this was rescued in the presence of CHIR‐99021, confirming that the effect of inhibition of GSK3α/β on platelet activation is integrin‐independent (Fig. S5C). This is consistent with the observation in Fig. S1A that activation of Akt and MAPK by CLEC‐2 is also integrin‐independent (Fig. S1A).

## Discussion

In this study, we have demonstrated that CLEC‐2 activates the PKC, PI3K–Akt and ERK pathways, leading to phosphorylation of GSK3α/β at its negative regulatory site, and loss of its inhibitory action, thereby enhancing platelet activation at low concentrations of rhodocytin. Akt regulates phosphorylation of GSK3α/β at 90 s and 300 s, whereas ERK only regulates phosphorylation at the latter time. This study therefore adds to the growing evidence that GSK3α/β mediates a weak, constitutive inhibitory signal that regulates platelet activation by threshold agonist concentrations, and that this is inhibited by PKC‐mediated, Akt‐mediated and MAPK‐mediated phosphorylation of GSK3α/β, with the effect of PKC being mediated in part through secretion of ADP.

The contribution of GSK3α/β to platelet activation has been controversial, as it appears to vary between agonists and is dependent on experimental conditions. For example, in the present study we showed that inhibition of GSK3α/β had a minimal effect on platelet activation by CRP, whereas collagen‐mediated activation was significantly reduced, as previously reported 13, 15, 37. This difference may be related to the much greater feedback role of ADP and TxA_2_ in platelet activation by collagen than in platelet activation by CRP, which may accentuate the effect of other mechanisms of platelet regulation. Alternatively, collagen may regulate GSK3α/β downstream of α_2_β_1_, which is not activated by CRP. A concern regarding pharmacologic inhibitors is that of off‐target effects. In consideration of this, we have shown that two structurally different inhibitors of GSK3α/β potentiate platelet aggregation, thereby strengthening the argument that GSK3α/β is a negative regulator of CLEC‐2 signaling but that it has a negligible effect on GPVI signaling.

The explanation for the differential time course of phosphorylation of GSK3α/β by rhodocytin, with Akt inducing significant phosphorylation at both 90 s and 300 s, and ERK inducing it at later times, is not known. However, it is noteworthy that, although not significant, a clear trend towards reduction in GSK3α/β phosphorylation was seen at 90 s in the presence of PD0325901, and this may explain why inhibition of ERK activation by PD0325901 also reduces platelet aggregation by CLEC‐2 stimulation. In line with these results, ERK has also been shown to phosphorylate GSK3α/β in other cells 38, 39. Moreover, inhibition of GSK3α/β can rescue platelets from the inhibitory action of Akt and MEK inhibitors with or without integrin activation, confirming GSK3α/β as a functional effector that lies downstream of Akt and ERK upon CLEC‐2 activation. In contrast, we were unable to rescue the inhibitory effect of the PI3K inhibitor wortmannin with a GSK3α/β inhibitor, suggesting that PI3K regulates additional pathways to those regulated by Akt. Consistent with this, wortmannin also inhibits aggregation at high concentrations of rhodocytin, whereas MK2206 only inhibits aggregation at low concentrations of this agonist. In this study, we have also shown that the PKC inhibitor Ro31‐8220 significantly reduces GSK3α/β phosphorylation independently of Akt and that this could not be fully rescued by the addition of ADP. This shows that PKC mediates phosphorylation of GSK3α/β through direct and indirect (i.e. ADP and Akt regulation) mechanisms, as previously reported for thrombin stimulation 13.

The molecular mechanism through which GSK3α/β regulates CLEC‐2‐mediated platelet activation could be through phosphorylation of its substrate, MAP‐tau. Inhibition of GSK3β leads to a reduction in MAP‐tau phosphorylation in thrombin‐simulated platelets 40. MAP‐tau has been shown to play a critical role in microtubule polymerization in other cells 41, 42. GSK3α/β might therefore regulate granule secretion in platelets through microtubule polymerization.

To date, there have been opposing reports on the importance of MAPK in platelets. The p38 inhibitor SB203580 has been reported to inhibit platelet aggregation induced by low concentrations of U46619 and collagen, although it is unclear whether this is attributable to off‐target effects on cyclooxygenase 43. Later, it was shown that a second p38 inhibitor, SB202190, also inhibits platelet activation 44. Several laboratories have reported that MEK inhibitors suppress collagen‐mediated, thrombin‐mediated, von Willebrand factor‐mediated and ADP‐mediated platelet activation 25, 44, 45, 46, whereas others have reported no effect 27. In our study, we used the selective MEK inhibitor PD0325901, and showed that ERK inhibition inhibits platelet activation in response to low concentrations of both rhodocytin and CRP, although this effect is readily overcome at higher concentrations of both ligands.

We have previously shown that, together, secondary mediators such as ADP and TxA_2_ are essential for robust activation of CLEC‐2 6. Consistent with this, we have shown that ADP and TxA_2_ regulate phosphorylation of Akt and ERK by CLEC‐2. In our previous study 6, we used 30 nm rhodocytin, and found complete blockade of CLEC‐2 activation in the presence of apyrase and indomethacin. In the present study, we observed a weak but significant increase in phosphorylation of Syk and PLCγ2 in response to a 10‐fold higher concentration of rhodocytin (300 nm). This demonstrates that the initial weak activation of CLEC‐2 by the snake toxin is significantly enhanced by the feedback action of ADP and TxA_2_. Furthermore, we have shown that blocking the effect of secreted ADP by apyrase or of secretion by Ro31‐8220 has a pronounced effect on Akt phosphorylation at 300 s but not at 90 s, which indicates two pathways of regulation, one of which is mediated by secretion of ADP. As CLEC‐2 activation is markedly inhibited in the presence of apyrase and indomethacin, however, we cannot readily compare the importance of CLEC‐2 and ADP in the regulation of Akt and MAPK phosphorylation. However, it noteworthy that, whereas ADP and TxA_2_ are important for reinforcing CLEC‐2 activation, neither ADP nor TxA_2_ induces CLEC‐2 phosphorylation 6.

In conclusion, we have shown that the Akt and MAPK pathways play a critical role in platelet activation at threshold levels of CLEC‐2 activation through phosphorylation of GSK3α/β, thus blocking its inhibitory action. PKC also regulates GSK3α/β phosphorylation at early times, independently of Akt.

## Addendum

A. J. Moroi designed and performed experiments, analyzed data, created the figures, and wrote the manuscript. S. P. Watson designed experiments, wrote the manuscript, and supervised the work.

## Disclosure of Conflict of Interests

The authors state that they have no conflict of interest.

## Supporting information


**Fig. S1.** Akt and MAPKs are activated after CLEC‐2 stimulation downstream of Syk and Src activation.
**Fig. S2.** The PI3K–Akt and MAPK pathways are activated after GPVI stimulation.
**Fig. S3.** The effects of PI3K, PKC, Akt and MAPK inhibition on GPVI‐mediated platelet aggregation.
**Fig. S4.** The effects of GSK3α/β inhibitors on CLEC‐2‐mediated platelet activation.
**Fig. S5.** GSK3α/β has a minimal effect on GPVI‐mediated platelet activation.

## References

[1] Colonna M , Samaridis J , Angman L . Molecular characterization of two novel C‐type lectin‐like receptors, one of which is selectively expressed in human dendritic cells. Eur J Immunol 2000; 30: 697–704. 10671229 10.1002/1521-4141(200002)30:2<697::AID-IMMU697>3.0.CO;2-M

[2] Kerrigan AM , Dennehy KM , Mourao‐Sa D , Faro‐Trindade I , Willment JA , Taylor PR , Eble JA , Reis e Sousa C , Brown GD . CLEC‐2 is a phagocytic activation receptor expressed on murine peripheral blood neutrophils. J Immunol 2009; 182: 4150–7. 19299712 10.4049/jimmunol.0802808 PMC2727695

[3] Sobanov Y , Bernreiter A , Derdak S , Mechtcheriakova D , Schweighofer B , Duchler M , Kalthoff F , Hofer E . A novel cluster of lectin‐like receptor genes expressed in monocytic, dendritic and endothelial cells maps close to the NK receptor genes in the human NK gene complex. Eur J Immunol 2001; 31: 3493–503. 11745369 10.1002/1521-4141(200112)31:12<3493::aid-immu3493>3.0.co;2-9

[4] Astarita JL , Acton SE , Turley SJ . Podoplanin: emerging functions in development, the immune system, and cancer. Front Immunol 2012; 3: 283. 22988448 10.3389/fimmu.2012.00283 PMC3439854

[5] Watson SP , Herbert JM , Pollitt AY . GPVI and CLEC‐2 in hemostasis and vascular integrity. J Thromb Haemost 2010; 8: 1456–67. 20345705 10.1111/j.1538-7836.2010.03875.x

[6] Pollitt AY , Grygielska B , Leblond B , Desire L , Eble JA , Watson SP . Phosphorylation of CLEC‐2 is dependent on lipid rafts, actin polymerization, secondary mediators, and Rac. Blood 2010; 115: 2938–46. 20154214 10.1182/blood-2009-12-257212

[7] Severin S , Pollitt AY , Navarro‐Nunez L , Nash CA , Mourao‐Sa D , Eble JA , Senis YA , Watson SP . Syk‐dependent phosphorylation of CLEC‐2: a novel mechanism of hem‐immunoreceptor tyrosine‐based activation motif signaling. J Biol Chem 2011; 286: 4107–16. 21098033 10.1074/jbc.M110.167502 PMC3039337

[8] Hughes CE , Auger JM , McGlade J , Eble JA , Pearce AC , Watson SP . Differential roles for the adapters Gads and LAT in platelet activation by GPVI and CLEC‐2. J Thromb Haemost 2008; 6: 2152–9. 18826392 10.1111/j.1538-7836.2008.03166.x PMC2710801

[9] Hughes CE , Pollitt AY , Mori J , Eble JA , Tomlinson MG , Hartwig JH , O'Callaghan CA , Futterer K , Watson SP . CLEC‐2 activates Syk through dimerization. Blood 2010; 115: 2947–55. 20154219 10.1182/blood-2009-08-237834 PMC4361903

[10] Suzuki‐Inoue K , Fuller GL , Garcia A , Eble JA , Pohlmann S , Inoue O , Gartner TK , Hughan SC , Pearce AC , Laing GD , Theakston RD , Schweighoffer E , Zitzmann N , Morita T , Tybulewicz VL , Ozaki Y , Watson SP . A novel Syk‐dependent mechanism of platelet activation by the C‐type lectin receptor CLEC‐2. Blood 2006; 107: 542–9. 16174766 10.1182/blood-2005-05-1994

[11] Jackson SP , Yap CL , Anderson KE . Phosphoinositide 3‐kinases and the regulation of platelet function. Biochem Soc Trans 2004; 32: 387–92. 15046614 10.1042/bst0320387

[12] Kroner C , Eybrechts K , Akkerman JW . Dual regulation of platelet protein kinase B. J Biol Chem 2000; 275: 27790–8. 10874027 10.1074/jbc.M000540200

[13] Moore SF , van den Bosch MT , Hunter RW , Sakamoto K , Poole AW , Hers I . Dual regulation of glycogen synthase kinase 3 (GSK3)alpha/beta by protein kinase C (PKC)alpha and Akt promotes thrombin‐mediated integrin alphaIIbbeta3 activation and granule secretion in platelets. J Biol Chem 2013; 288: 3918–28. 23239877 10.1074/jbc.M112.429936 PMC3567645

[14] Woulfe D , Jiang H , Morgans A , Monks R , Birnbaum M , Brass LF . Defects in secretion, aggregation, and thrombus formation in platelets from mice lacking Akt2. J Clin Invest 2004; 113: 441–50. 14755341 10.1172/JCI20267 PMC324545

[15] O'Brien KA , Stojanovic‐Terpo A , Hay N , Du X . An important role for Akt3 in platelet activation and thrombosis. Blood 2011; 118: 4215–23. 21821713 10.1182/blood-2010-12-323204 PMC3204738

[16] Chen J , De S , Damron DS , Chen WS , Hay N , Byzova TV . Impaired platelet responses to thrombin and collagen in AKT‐1‐deficient mice. Blood 2004; 104: 1703–10. 15105289 10.1182/blood-2003-10-3428 PMC1569945

[17] Dangelmaier C , Manne BK , Liverani E , Jin J , Bray P , Kunapuli SP . PDK1 selectively phosphorylates Thr(308) on Akt and contributes to human platelet functional responses. Thromb Haemost 2014; 111: 508–17. 24352480 10.1160/TH13-06-0484 PMC4079046

[18] Laurent PA , Severin S , Gratacap MP , Payrastre B . Class I PI 3‐kinases signaling in platelet activation and thrombosis: PDK1/Akt/GSK3 axis and impact of PTEN and SHIP1. Adv Biol Regul 2014; 54: 162–74. 24095650 10.1016/j.jbior.2013.09.006

[19] Chen X , Zhang Y , Wang Y , Li D , Zhang L , Wang K , Luo X , Yang Z , Wu Y , Liu J . PDK1 regulates platelet activation and arterial thrombosis. Blood 2013; 121: 3718–26. 23444402 10.1182/blood-2012-10-461897

[20] Moore SF , Hunter RW , Hers I . mTORC2 protein complex‐mediated Akt (protein kinase B) serine 473 phosphorylation is not required for Akt1 activity in human platelets [corrected]. J Biol Chem 2011; 286: 24553–60. 21592956 10.1074/jbc.M110.202341 PMC3137030

[21] Resendiz JC , Kroll MH , Lassila R . Protease‐activated receptor‐induced Akt activation – regulation and possible function. J Thromb Haemost 2007; 5: 2484–93. 17883592 10.1111/j.1538-7836.2007.02769.x

[22] Deb TB , Coticchia CM , Dickson RB . Calmodulin‐mediated activation of Akt regulates survival of c‐Myc‐overexpressing mouse mammary carcinoma cells. J Biol Chem 2004; 279: 38903–11. 15247222 10.1074/jbc.M405314200

[23] Yano S , Tokumitsu H , Soderling TR . Calcium promotes cell survival through CaM‐K kinase activation of the protein‐kinase‐B pathway. Nature 1998; 396: 584–7. 9859994 10.1038/25147

[24] Hayashi H , Sudo T . Effects of the cAMP‐elevating agents cilostamide, cilostazol and forskolin on the phosphorylation of Akt and GSK‐3beta in platelets. Thromb Haemost 2009; 102: 327–35. 19652884 10.1160/TH08-12-0781

[25] Toth‐Zsamboki E , Oury C , Cornelissen H , De Vos R , Vermylen J , Hoylaerts MF . P2X1‐mediated ERK2 activation amplifies the collagen‐induced platelet secretion by enhancing myosin light chain kinase activation. J Biol Chem 2003; 278: 46661–7. 14500714 10.1074/jbc.M308452200

[26] Flevaris P , Li Z , Zhang G , Zheng Y , Liu J , Du X . Two distinct roles of mitogen‐activated protein kinases in platelets and a novel Rac1‐MAPK‐dependent integrin outside‐in retractile signaling pathway. Blood 2009; 113: 893–901. 18957688 10.1182/blood-2008-05-155978 PMC2630274

[27] Borsch‐Haubold AG , Kramer RM , Watson SP . Inhibition of mitogen‐activated protein kinase kinase does not impair primary activation of human platelets. Biochem J 1996; 318(Pt 1): 207–12. 8761473 10.1042/bj3180207 PMC1217609

[28] Borsch‐Haubold AG , Pasquet S , Watson SP . Direct inhibition of cyclooxygenase‐1 and ‐2 by the kinase inhibitors SB 203580 and PD 98059. SB 203580 also inhibits thromboxane synthase. J Biol Chem 1998; 273: 28766–72. 9786874 10.1074/jbc.273.44.28766

[29] Eble JA , Beermann B , Hinz HJ , Schmidt‐Hederich A . alpha2beta 1 integrin is not recognized by rhodocytin but is the specific, high affinity target of rhodocetin, an RGD‐independent disintegrin and potent inhibitor of cell adhesion to collagen. J Biol Chem 2001; 276: 12274–84. 11121411 10.1074/jbc.M009338200

[30] Hughes CE , Sinha U , Pandey A , Eble JA , O'Callaghan CA , Watson SP . Critical role for an acidic amino acid region in platelet signaling by the HemITAM (hemi‐immunoreceptor tyrosine‐based activation motif) containing receptor CLEC‐2 (C‐type lectin receptor‐2). J Biol Chem 2013; 288: 5127–35. 23264619 10.1074/jbc.M112.411462 PMC3576117

[31] Fuller GL , Williams JA , Tomlinson MG , Eble JA , Hanna SL , Pohlmann S , Suzuki‐Inoue K , Ozaki Y , Watson SP , Pearce AC . The C‐type lectin receptors CLEC‐2 and Dectin‐1, but not DC‐SIGN, signal via a novel YXXL‐dependent signaling cascade. J Biol Chem 2007; 282: 12397–409. 17339324 10.1074/jbc.M609558200 PMC1997429

[32] Mocsai A , Ruland J , Tybulewicz VL . The SYK tyrosine kinase: a crucial player in diverse biological functions. Nat Rev Immunol 2010; 10: 387–402. 20467426 10.1038/nri2765 PMC4782221

[33] Kim S , Mangin P , Dangelmaier C , Lillian R , Jackson SP , Daniel JL , Kunapuli SP . Role of phosphoinositide 3‐kinase beta in glycoprotein VI‐mediated Akt activation in platelets. J Biol Chem 2009; 284: 33763–72. 19700402 10.1074/jbc.M109.048553 PMC2797145

[34] Gilio K , Munnix IC , Mangin P , Cosemans JM , Feijge MA , van der Meijden PE , Olieslagers S , Chrzanowska‐Wodnicka MB , Lillian R , Schoenwaelder S , Koyasu S , Sage SO , Jackson SP , Heemskerk JW . Non‐redundant roles of phosphoinositide 3‐kinase isoforms alpha and beta in glycoprotein VI‐induced platelet signaling and thrombus formation. J Biol Chem 2009; 284: 33750–62. 19815551 10.1074/jbc.M109.048439 PMC2797144

[35] Inoue O , Suzuki‐Inoue K , Dean WL , Frampton J , Watson SP . Integrin alpha2beta1 mediates outside‐in regulation of platelet spreading on collagen through activation of Src kinases and PLCgamma2. J Cell Biol 2003; 160: 769–80. 12615912 10.1083/jcb.200208043 PMC2173361

[36] Yanaga F , Poole A , Asselin J , Blake R , Schieven GL , Clark EA , Law CL , Watson SP . Syk interacts with tyrosine‐phosphorylated proteins in human platelets activated by collagen and cross‐linking of the Fc gamma‐IIA receptor. Biochem J 1995; 311(Pt 2): 471–8. 7487883 10.1042/bj3110471 PMC1136023

[37] Barry FA , Graham GJ , Fry MJ , Gibbins JM . Regulation of glycogen synthase kinase 3 in human platelets: a possible role in platelet function? FEBS Lett 2003; 553: 173–8. 14550568 10.1016/s0014-5793(03)01015-9

[38] Miki T , Miura T , Hotta H , Tanno M , Yano T , Sato T , Terashima Y , Takada A , Ishikawa S , Shimamoto K . Endoplasmic reticulum stress in diabetic hearts abolishes erythropoietin‐induced myocardial protection by impairment of phospho‐glycogen synthase kinase‐3beta‐mediated suppression of mitochondrial permeability transition. Diabetes 2009; 58: 2863–72. 19755525 10.2337/db09-0158 PMC2780889

[39] Ding Q , Xia W , Liu JC , Yang JY , Lee DF , Xia J , Bartholomeusz G , Li Y , Pan Y , Li Z , Bargou RC , Qin J , Lai CC , Tsai FJ , Tsai CH , Hung MC . Erk associates with and primes GSK‐3beta for its inactivation resulting in upregulation of beta‐catenin. Mol Cell 2005; 19: 159–70. 16039586 10.1016/j.molcel.2005.06.009

[40] Li D , August S , Woulfe DS . GSK3beta is a negative regulator of platelet function and thrombosis. Blood 2008; 111: 3522–30. 18218855 10.1182/blood-2007-09-111518 PMC2275019

[41] Cho JH , Johnson GV . Glycogen synthase kinase 3beta phosphorylates tau at both primed and unprimed sites. Differential impact on microtubule binding. J Biol Chem 2003; 278: 187–93. 12409305 10.1074/jbc.M206236200

[42] Avila J , Lucas JJ , Perez M , Hernandez F . Role of tau protein in both physiological and pathological conditions. Physiol Rev 2004; 84: 361–84. 15044677 10.1152/physrev.00024.2003

[43] Saklatvala J , Rawlinson L , Waller RJ , Sarsfield S , Lee JC , Morton LF , Barnes MJ , Farndale RW . Role for p38 mitogen‐activated protein kinase in platelet aggregation caused by collagen or a thromboxane analogue. J Biol Chem 1996; 271: 6586–9. 8636072 10.1074/jbc.271.12.6586

[44] Li Z , Zhang G , Feil R , Han J , Du X . Sequential activation of p38 and ERK pathways by cGMP‐dependent protein kinase leading to activation of the platelet integrin alphaIIb beta3. Blood 2006; 107: 965–72. 16210341 10.1182/blood-2005-03-1308 PMC1464421

[45] Garcia A , Quinton TM , Dorsam RT , Kunapuli SP . Src family kinase‐mediated and Erk‐mediated thromboxane A2 generation are essential for VWF/GPIb‐induced fibrinogen receptor activation in human platelets. Blood 2005; 106: 3410–14. 16020504 10.1182/blood-2005-05-1933 PMC1895051

[46] Garcia A , Shankar H , Murugappan S , Kim S , Kunapuli SP . Regulation and functional consequences of ADP receptor‐mediated ERK2 activation in platelets. Biochem J 2007; 404: 299–308. 17298299 10.1042/BJ20061584 PMC1868805

