# Supplementary material for: Akt and mitogen‐activated protein kinase enhance C‐type lectin‐like receptor 2‐mediated platelet activation by inhibition of glycogen synthase kinase 3α/β
Source: J Thromb Haemost. 2015 May 9;13(6):1139–50. doi: 10.1111/jth.12954 (PMC4737230; doi:10.1111/jth.12954)
Supplement: Supplementary file 1 — Fig. S1. Akt and MAPKs are activated after CLEC‐2 stimulation downstream of Syk and Src activation. Fig. S2. The PI3K–Akt and MAPK pathways are activated after GPVI stimulation. Fig. S3. The effects of PI3K, PKC, Akt and MAPK inhibition on GPVI‐mediated platelet aggregation. Fig. S4. The effects of GSK3α/β inhibitors on CLEC‐2‐mediated platelet activation. Fig. S5. GSK3α/β has a minimal effect on GPVI‐mediated platelet activation. [file JTH-13-1139-s001.docx]

**Supplementary figures**


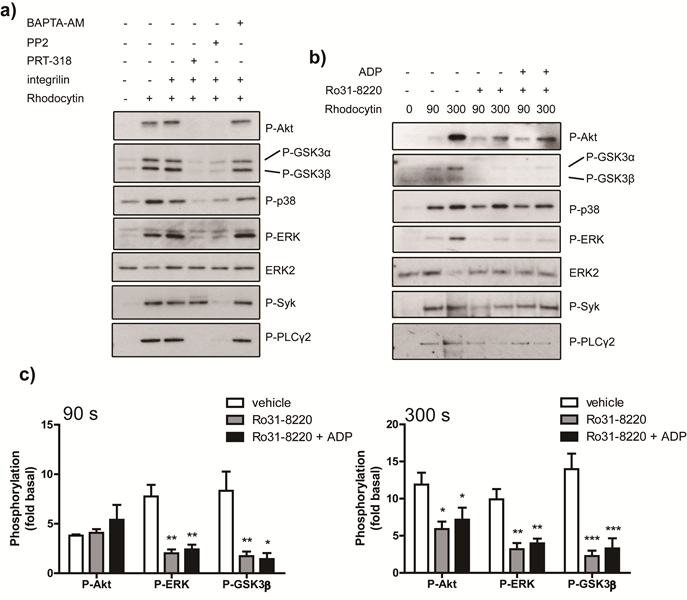


**Fig. S1.** Akt and MAPKs are activated after CLEC-2 stimulation downstream of Syk and Src activation. (a) Washed human platelets (5×10^8^ mL^-1^) were pre-incubated with 10 μM PP2, 5 μM PRT-318, 10 μM BAPTA-AM or DMSO (vehicle control). Platelets were stimulated with 300 nM rhodocytin in the absence or presence of integrilin (9 μM) for 300 s before extraction. (b) Washed human platelets were pre-treated with integrilin (9 µM). Platelets were pre-incubated with 5 µM Ro31-8220 or DMSO (vehicle control) and then stimulated with 300 nM rhodocytin in the absence or presence of ADP (10 μM) for indicated times before extraction. Whole cell lysates were analyzed for phosphorylation of Akt (Thr 308), p38 (Thr 180/182), ERK (Thr 202/Tyr 204), GSK3α/β (Ser 21/9), Syk (Tyr352) and PLCγ2 (Tyr1217) by western blotting. ERK2 was used as a loading control. The blot shown is representative of three independent experiments. (c) Densitometric measurements of phospho-Akt, phospho-ERK and phospho-GSK3β at 90 s (left) and 300 s (right) after stimulation are expressed as fold increase over the basal control (DMSO, no rhodocytin). The blot shown is representative of three independent experiments. *P < 0.05, **P < 0.01, ***P < 0.001, compared with the vehicle control.


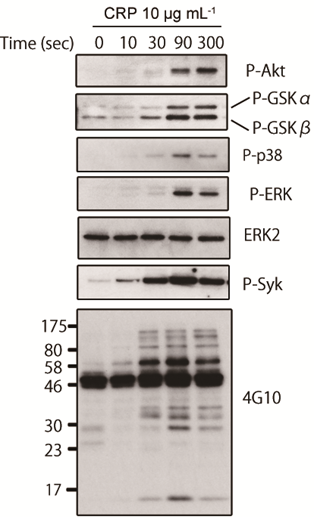


**Fig. S2.** PI3K/Akt and MAPK pathways are activated after GPVI stimulation. Washed human platelets (5×10^8^ mL^-1^) were pre-treated with integrilin (9 μM). Platelets were pre-incubated with DMSO (vehicle control) and then stimulated with 10 μg mL^-1^ CRP for the indicated times before extraction. Whole cell lysates were analyzed for phosphorylation of Akt (Thr 308), p38 (Thr 180/182), ERK (Thr 202/Tyr 204), GSK3α/β (Ser 21/9) and Syk (Tyr 352) by western blotting. Total tyrosine phosphorylation was detected by 4G10 mAb and ERK2 was used as a loading control. The blot shown is representative of three independent experiments.


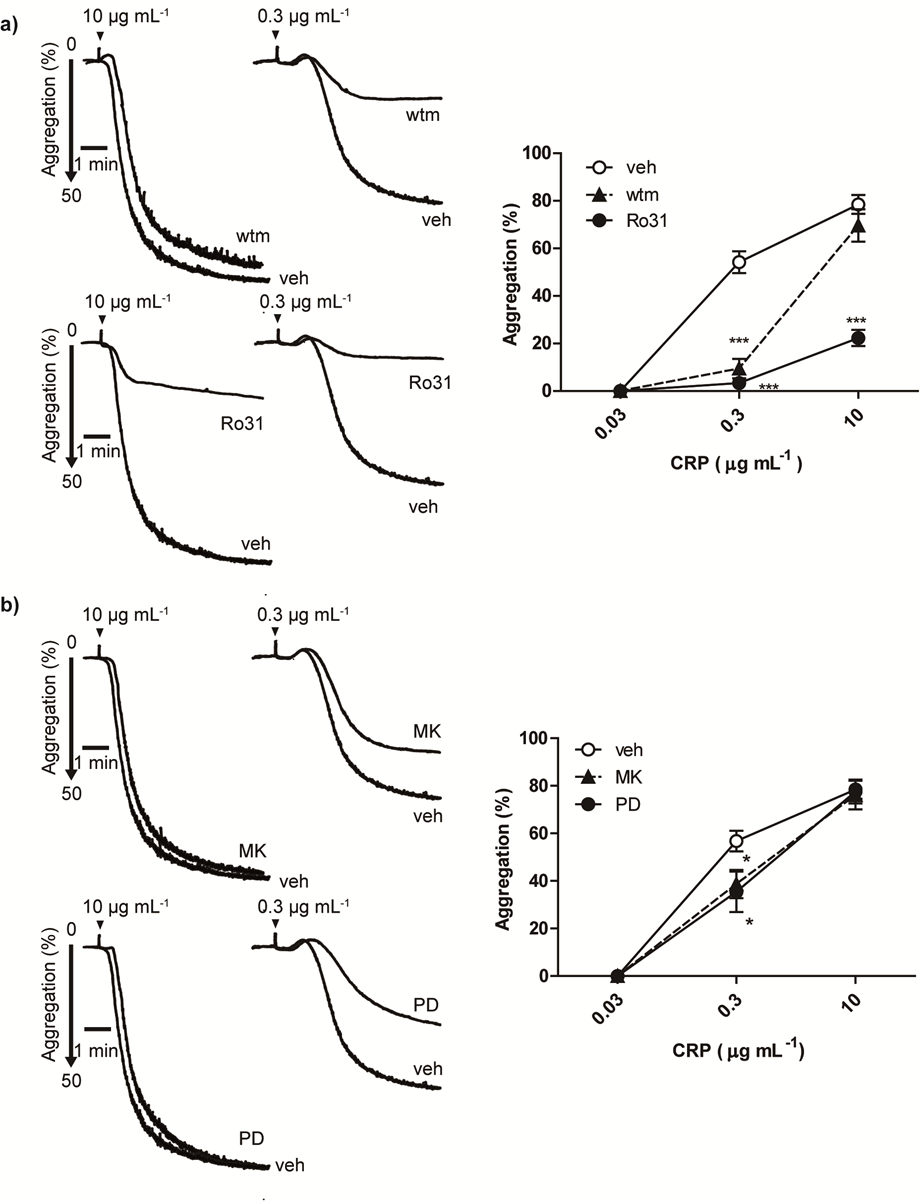


**Fig. S3.** The effect of PI3K, PKC, Akt and MAPK inhibition on GPVI-mediated platelet aggregation. Washed human platelets (2×10^8^ mL^-1^) were pre-incubated with 100 nM wortmannin (wtm), 5 µM Ro31-8220 (Ro31) (a), 1 μM MK2206 (MK), 5 μM PD0325901 (PD) (b) or DMSO (vehicle control, veh) and then stimulated with the indicated dose of CRP at 37°C and platelet aggregation were measured for 5 min. The left-side figures show the aggregometer tracings, each being representative of results obtained from five different donors and experiments. The right-side graphs shows the effect of wortmannin, Ro31-8220, MK2206 or PD0325901on aggregation at 5 min; data bars are each the mean± S.E.M. of n = 5 experiments. **P* < 0.05, ****P* < 0.001, compared with the vehicle control (DMSO).


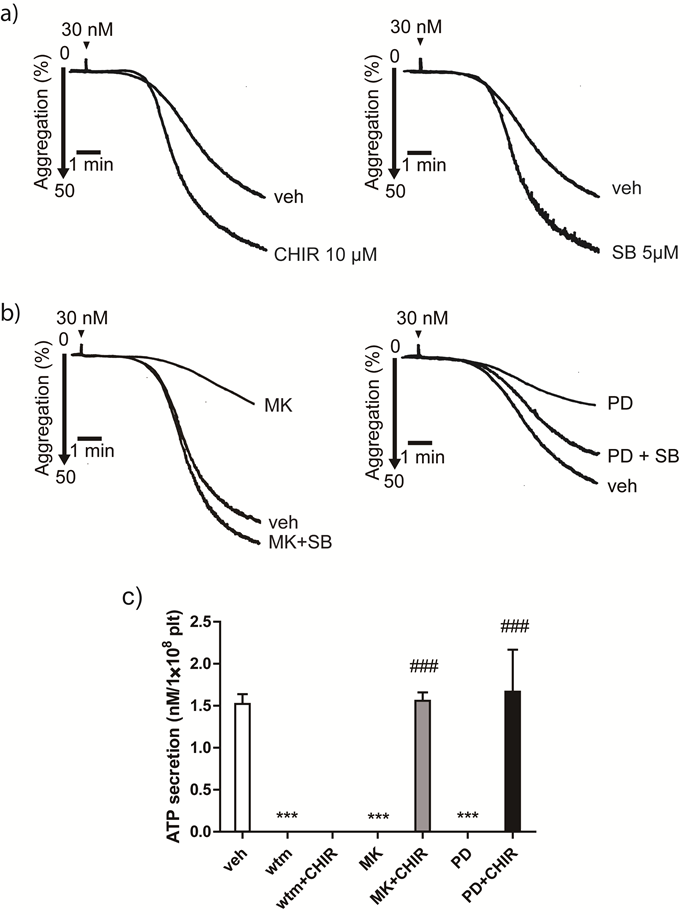


**Fig. S4.** The effect of GSK3α/β inhibitors on CLEC-2-mediated platelet activation. Washed human platelets (2×10^8^ mL^-1^) were pre-incubated with 10 µM CHIR-99021 (CHIR), 5 µM SB216763 (SB) or DMSO (vehicle control, veh) (a) 1 µM MK2206 (MK), 5 µM PD0325901 (PD) in the presence or absence of 5 µM SB216763 or DMSO (vehicle control) (b) and then stimulated with 30 nM rhodocytin at 37°C and platelet aggregation were measured for 5 min. Each aggregometer tracing is representative of results obtained from three different donors and experiments. (c) Quantification of peak platelet ATP release (mean ± S.E.M., n = 3–5) in the presence of the vehicle, MK2206 or PD0325901 and CHIR-99021 in platelets activated by 10 nM rhodocytin. ****P* < 0.001, compared with the vehicle control (DMSO), ### *P* < 0.001, compared with MK2206 or PD0325901 single treatment.

**
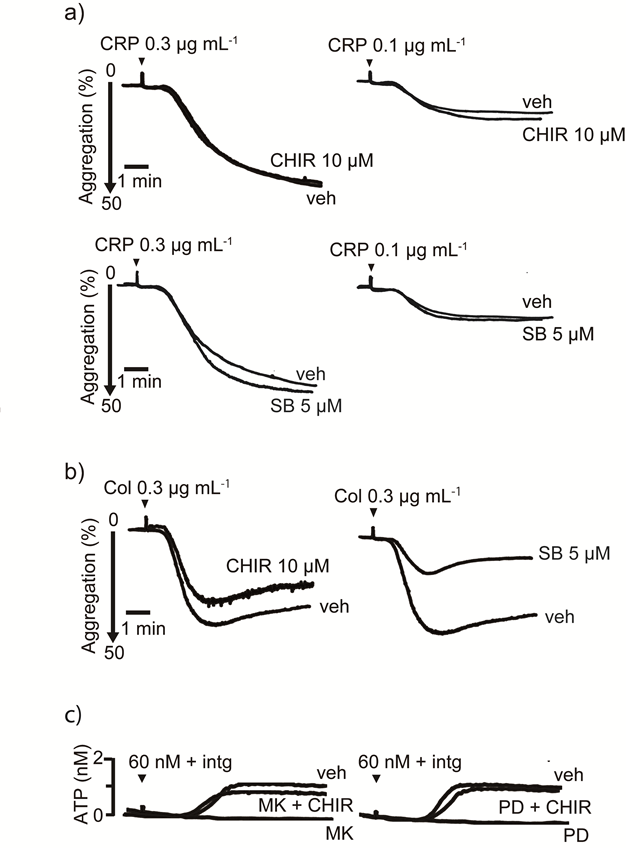
**

**Fig. S5.** GSK3α/β has a minimal effect on GPVI-mediated platelet activation.

Washed human platelets (2×10^8^ mL^-1^) were pre-incubated with 10 µM CHIR-99021 (CHIR), 5 µM SB216763 (SB) or DMSO (vehicle control, veh) and then stimulated with 0.3 µg mL^-1^ or 0.1 µg mL^-1^ CRP (a) and 0.3 µg mL^-1^ collagen (b) at 37°C and platelet aggregation were measured for 5 min. (c) Washed human platelets (2×10^8^ mL^-1^) were pre-treated with 9µM integrilin (intg). Platelets were then pre-incubated with 1 µM MK2206 (MK), 5 µM PD0325901 (PD) in the presence or absence of 10 µM CHIR99021 or DMSO (vehicle control) and then stimulated with 60 nM rhodocytin at 37°C and platelet ATP release were measured for 5 min. Each aggregometer tracing is representative of results obtained from three different donors and experiments.
